# Canine vector-borne infections in Mauritius

**DOI:** 10.1186/s13071-015-0790-9

**Published:** 2015-03-23

**Authors:** Gary Kwok Cheong Lee, Jean Alain Ean Ignace, Ian Duncan Robertson, Peter John Irwin

**Affiliations:** Vector and Water-Borne Pathogen Research Group, School of Veterinary and Life Sciences, Murdoch University, Murdoch, WA 6150 Australia; Sir Seewoosagur Ramgoolam Animal Hospital, Mauritius Society for Animal Welfare, Rose Hill, Mauritius

**Keywords:** Mauritius, *Ehrlichia*, *Anaplasma*, *Dirofilaria immitis*, *Hepatozoon canis*, *Rhipicephalus sanguineus*, *Amblyomma variegatum*, Canine vector-borne diseases

## Abstract

**Background:**

Canine vector-borne diseases have a worldwide distribution, but to the best of our knowledge, no research has been carried out to evaluate their presence on the Indian Ocean island of Mauritius. An investigation into canine vector-borne infections was conducted in dogs (n = 78) resident at an animal shelter in Port Louis, Mauritius using a combination of traditional microscopy and serological methods.

**Methods:**

Ticks were manually collected from the stray dog population for identification as well as for quantifying tick burdens. Blood was also collected from each dog via either the jugular vein or the cephalic vein, and was stored in EDTA tubes. The stored blood was then used to measure PCV values, make blood smears for the identification of parasites, and used for serological testing of vector-borne disease.

**Results:**

A total of 178 ticks were collected from 52 dogs and identified as *Rhipicephalus sanguineus* (175/178) or *Amblyomma variegatum* (3/178). Twenty-six (33%; 95% CI 23, 45) dogs were seropositive for *Ehrlichia* spp*.*, and 12 (15%; 95% CI 8, 25) for *Anaplasma* spp., *Dirofilaria* antigen was detected in 14 (18%; 95% CI 10, 28), and nine (12%; 95% CI 5, 21) dogs had *Hepatozoon canis* gamonts observed in blood films during microscopic examination. Eleven (14%; 95% CI 7, 24) dogs were co-infected with two pathogens. *Borrelia burgdorferi* antibodies were not detected in any dogs.

**Conclusions:**

Infection with these pathogens had no significant effect on the packed cell volume (PCV), but high tick burdens were significantly associated with the presence of a tick-borne pathogen. This is the first study of its kind on the dog population in Mauritius and demonstrates the presence of previously undocumented canine vector-borne infections on the island. The relatively high proportion of infected dogs within the study should alert clinicians to the presence of canine vector-borne diseases on the island of Mauritius.

## Background

Mauritius is an island of volcanic origin that lies within the South-West Indian Ocean (20°17′S and 57°33′E). The climate is tropical with hot and humid summers and warm and drier winters. Average temperatures at sea level are moderately high (22-31°C) and decrease with increasing altitude to a minimum of 12°C (maximum altitude 904 m) [1]. The mean humidity around the island is generally >80% but rainfall varies significantly between regions, with approximately 5000 mm/year on the high grounds and only 1000 mm/year in the coastal regions. Domestic dogs were introduced to the island by Dutch settlers in the 17th Century, and many dogs nowadays roam the island as strays. The large numbers of stray dogs together with favourable climatic conditions are ideal to facilitate the transmission of vector-borne pathogens.

Canine ehrlichiosis, anaplasmosis, hepatozoonosis and dirofilariasis are vector-borne diseases with a worldwide distribution, yet to the best of our knowledge there have been no studies into the occurrence or prevalence of these diseases in dogs in Mauritius [1,2]. With the exception of dirofilariasis (heartworm disease) that is transmitted by mosquitoes, these diseases are transmitted by ticks, notably *Rhipicephalus sanguineus* (the brown dog tick), which itself has a ubiquitous geographical distribution and has been reported on Mauritius [1]. From a clinical perspective, vector-borne diseases traditionally pose a diagnostic challenge due to their non-specific symptomology and often sub-clinical nature, thus a high degree of diagnostic suspicion is required by veterinarians in order to make a diagnosis [3].

The objective of this investigation was to conduct a cross-sectional study using a combination of serological and traditional microscopic techniques to determine the presence of haemotropic vector-borne infections in a population of impounded dogs in Mauritius and, in addition, to identify the ticks infecting these dogs.

## Methods

### Study population and sample collection

Sampling was conducted at an animal shelter operated by the Mauritius Society for Animal Welfare (MSAW) in Port Louis, the capital city of Mauritius, in January 2014, with approval of the Murdoch University Animal Ethics Committee (Permit No. R2622/13). This shelter serves as the island’s main pound and receives stray dogs from all over the country. There are approximately 10–15 dogs impounded each day, including dogs of all ages (including puppies) and breeds. Dogs that are suffering from overt diseases are euthanized upon arrival. The other dogs are impounded for three days. The dogs that have not been reclaimed after three days are either euthanized or kept for rehoming at the MSAW headquarters in Rose Hill should they be in apparently good health.

Each dog was restrained and examined for ticks, which were collected by gentle manual removal and immediately placed in tubes containing 70% ethanol for later identification. Each dog was also given a tick burden score (low, medium or high) depending on the total number of visible ticks on the dog at the time of collection. Anatomical regions assessed were the head, ears, neck, thorax, abdomen, fore and hind limbs, interdigital areas, axilla, tail and inguinal area. A low score represented the presence of <10 ticks in all locations, a medium burden indicated 10 to 20 ticks, and for a high score >20 ticks were observed and recorded. Ticks were later examined under a dissecting stereo microscope (SZ61, Olympus, Japan) and identified to species level using standard keys [4,5].

A blood sample was collected from either the jugular or the cephalic vein, using standard technique. The blood was immediately transferred into 2.5 ml tubes containing ethylene diamine tetra acetic acid (EDTA); the tube was gently inverted to thoroughly mix the anticoagulant, and two blood smears were made on glass microscope slides within two hours of collection. Blood films were fixed in methanol immediately and later stained using a commercial available Romanowski staining system (Diff Quik, Harleco, USA).

### Serological testing, laboratory measurements and microscopic examination

Anticoagulated blood was used on the same day to detect antibodies to *Ehrlichia canis*, *Ehrlichia ewingii*, *Anaplasma phagocytophilum*, *A. platys*, and *Borrelia burgdorferi*, and antigen of *Dirofilaria immitis* using a cage-side immunochromatographic test (Snap 4Dx Plus, IDEXX laboratories, Westbrook, ME, USA), according to the manufacturer’s instructions. The packed cell volume (PCV) was measured using the EDTA-stored blood using a portable centrifuge (ZIPocrit, Shanghai LW Scientific Co. Ltd., Shanghai, China). The blood smears were evaluated using the technique described by Allison *et al*. [6]; forty medium power (X400) fields were evaluated for each smear.

### Statistical analysis

The data from PCV values and the presence of pathogens (*Ehrlichia*, *Anaplasma*, *Dirofilaria*, and *Hepatozoon* spp.) were coded and analysed with analysis of variance (ANOVA), after testing for homogeneity of variances with the Levene Statistic. A Pearson’s Chi-square test was used to assess the relationship between tick burden, presence of pathogens and gender. Co-infections with different pathogens were treated as an individual entity. All results were considered to be significant at p ≤ 0.05.

## Results

### Dogs

A total of 78 dogs were examined and sampled for inclusion in this study. These comprised 39 male and 39 females, of a variety of breeds and cross-breeds (data not shown).

### Ticks

**Figure 1:**
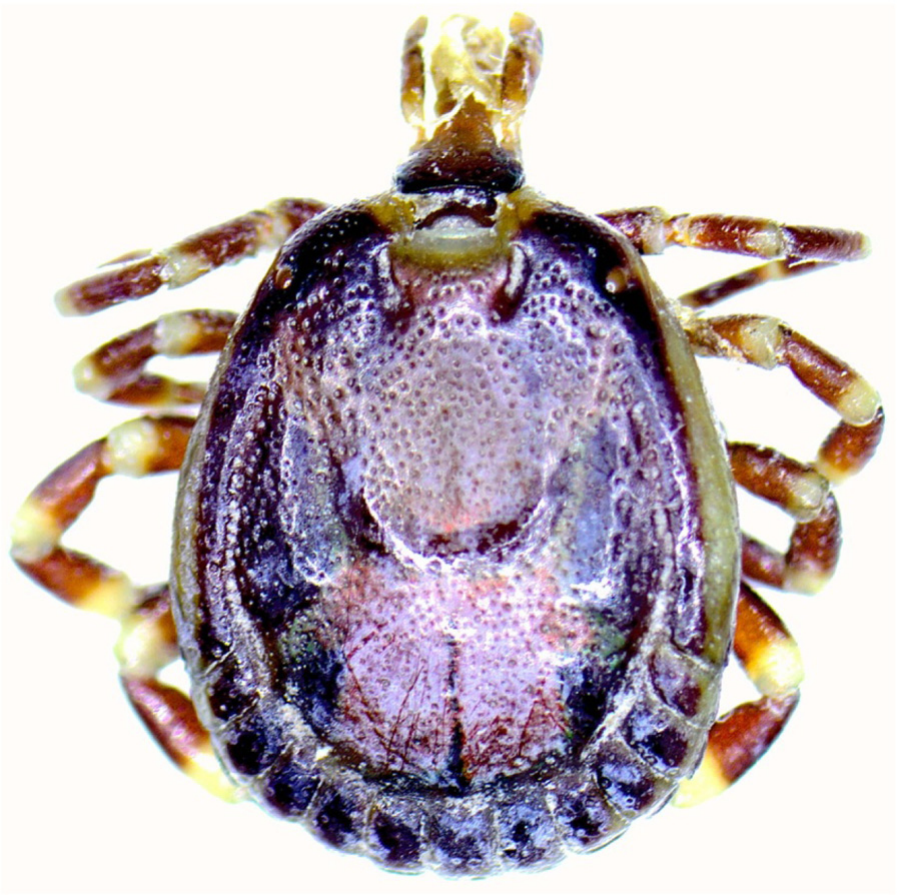
**Male** ***Amblyomma variegatum*** **tick.**

One hundred and seventy eight ticks (males n=73; females n=95; nymphs n=10) were collected from a total of 52 dogs. The majority (175/178) of ticks (98%; 95% CI 95, 100) were identified as *Rhipicephalus sanguineus*, and three (2%; 95% CI 0, 5) were *Amblyomma variegatum* (Figure 1). These *A. variegatum* ticks (1 male, 1 female, 1 nymph) were found on one dog only, together with a single male *Rhipicephalus sanguineus*.

### Serology and microscopy test results

**Figure 2:**
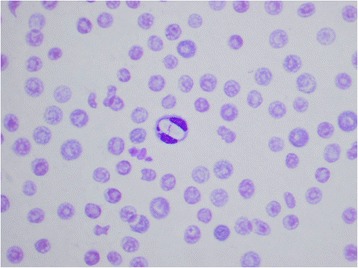
***Hepatozoon canis*** **gamont within a segmented neutrophil.** Note the artefacts within the red blood cells.

Antibodies to *Ehrlichia* spp. and *Anaplasma* spp. were detected in 26/78 (33%; 95% CI 23, 45), and 12/78 (15%; 95% CI 8, 25) dogs, respectively, and antigenaemia indicative of *Dirofilaria immitis* infection was detected in 14/78 dogs (18%; 95% CI 10, 28). None of the dogs tested positive for *Borrelia burgdorferi*, yet four (5%; 95% CI 1, 13) had co-infections with *Ehrlichia* and *Anaplasma* (Table 1). Unfortunately comprehensive blood smear assessment was not possible due to significant artefact which precluded evaluation of the red blood cells and platelets in all the smears made. This was thought retrospectively to be associated with an error in the fixation process. However, intracytoplasmic gamonts, consistent with *Hepatozoon canis*, were observed in neutrophils in the blood films of nine (12%; 95% CI 5, 21) of the dogs, including two dogs that were negative to all other pathogens (Figure 2). Of the 14 dogs that were positive to *D. immitis* antigen on serology, seven (50%; 95% CI 23, 77) had visible microfilariae on examination of the blood films. One dog had microfilariae visible on cytological examination of the blood smears, but was negative for *D. immitis* antigen.

**Table 1:** **Presence of infections in male and female dogs**

	***Ehrlichia spp.*** **only (serology)**	***Anaplasma spp.*** **only (serology)**	***Dirofilaria immitis*** **only (serology)**	***Borrelia burgdorferi*** **(serology)**	***Hepatozoon canis*** **only (cytology)**	***Ehrlichia spp.*** ** +** ***Anaplasma spp.*** **(serology)**	***Ehrlichia spp. + Hepatozoon canis*** **(serology + cytology)**	***Hepatozoon canis + Dirofilaria immitis*** **(serology + cytology)**	**Total**
Male	8	4	8	0	0	3	4	1	28
Female	9	4	4	0	2	1	1	1	22
Total	17	8	12	0	2	4	5	2	50

### Statistical data

Result from the ANOVA showed no significant difference in PCV between infection groups, and no significant difference in PCV in *Ehrlichia* spp. positive and negative groups (p=0.29), *Anaplasma* spp. positive and negative groups (p=0.43), and *D. immitis* positive and negative groups (p=0.40). However, animals which were positive for *H. canis* had significantly lower PCV values than those animals that did not harbour the pathogen (p=0.03) (Table 2). Tick burdens had no significant effect on PCV values (p=0.16).

**Table 2:** **Relationship between the presence of pathogens and PCV values**

		**n**	**Mean PCV ± SE**	**P value**
*E. canis*	Negative	52	44.50 ± 1.782	0.29
	Positive	26	40.92 ± 3.075	
*A. platys*	Negative	66	42.77 ± 1.770	0.43
	Positive	12	46.25 ± 3.050	
*H. canis*	Negative	69	44.54 ± 1.638	0.03*
	Positive	9	33.89 ± 4.244	
*D. immitis*	Negative	64	42.69 ± 1.734	0.40
	Positive	14	46.14 ± 1.569	
No of pathogens	0	28	43.79 ± 2.563	0.39
	1	39	44.46 ± 2.207	
	2	11	38.00 ± 4.507	

*Abbreviations*: *n* Total number of dogs, *PCV* Packed Cell Volume, *SE* Standard Error.

*- result was statistically significant.

Heavier tick burdens were significantly associated with higher numbers of pathogens diagnosed (co-infections were significantly associated with greater tick burdens) (p < 0.05). Greater tick burdens were also significantly associated with the presence of *E. canis* and *H. canis* (Table 3). Interestingly lower tick burdens were significantly associated with the presence of *D. immitis*. Tick burdens were not associated with positive serology for *Anaplasma* spp. and gender did not show any significant relationship with any of the other variables.

**Table 3:** **Relationship between tick burden and presence of pathogens**

		**Tick burden**				**Pearson’s Chi-square**
		**None**	**Low**	**Medium**	**High**	**Asymp. Sig (2-sided)**
*E. canis*	Negative	22	18	10	2	0.00*
	Positive	4	5	7	10	
*A. platys*	Negative	22	18	16	10	0.59
	Positive	4	5	1	2	
*H. canis*	Negative	24	23	14	8	0.02*
	Positive	2	0	3	4	
*D. immitis*	Negative	17	20	15	12	0.04*
	Positive	9	3	2	0	
No of pathogens	0	8	12	7	1	0.02*
	1	17	9	7	6	
	2	1	2	3	5	

*Abbreviations*: *Asymp. Sig* Asymptotic significance.

*- result was statistically significant.

## Discussion

The dogs that were tested during this study were free-roaming strays collected throughout the island of Mauritius and kept at the MSAW pound in Port-Louis. Since it took several days to collect all the samples, new batches of dogs were continuously arriving to be assessed. In this regard therefore, the results likely provide a representative snapshot of several canine vector-borne diseases amongst the stray population throughout this southern Indian Ocean island. However, it should be noted that this stray population was unlikely to be receiving ectoparasiticide prophylaxis and may therefore not be representative of the entire canine population on the island.

Not unexpectedly, given the environmental conditions and the hosts sampled, the majority of the ticks (98%) were *R. sanguineus*, the brown dog tick. *Rhipicephalus sanguineus* is the most widespread tick in the world and has been previously documented as being the most common tick found on dogs in Mauritius [1,7,8]. *Rhipicephalus sanguineus* is the main vector for *E. canis*, the cause of canine monocytic ehrlichiosis (CME), and is also responsible for the transmission of other pathogens to dogs including *Babesia canis vogeli*, *Hepatozoon canis*, and possibly *Anaplasma platys* [9]. In addition, in other parts of the world, *R. sanguineus* has been implicated as a vector for pathogens of human medical importance, notably Mediterranean fever (*Rickettsia conorii*) and other rickettsial infections (*Rickettsia massiliae* and *Rickettsia* rickettsia) [7,10]. The brown dog tick also serves as an intermediate host for the dog filarial parasites *Cercopithifilaria bainae* and *Cercopithifilaria grassii*, which were not tested for in this study [10,11].

Interestingly, three *A. variegatum* ticks were collected from a single dog. This tick is most commonly associated with cattle in Africa but has been known to feed on other animal species including dogs and people [1,12,13]. *Amblyomma variegatum* is widely distributed throughout Africa and has been reported previously in Mauritius, predominantly on cattle and deer [1,14,15]. Our results suggest that this tick is not commonly found in the Mauritian dog population; it is possible the dog was previously in contact with cattle or deer prior to being captured and taken to the shelter, but this was not possible to verify. *Amblyomma variegatum* is the principal vector responsible for the spread of cowdriosis (*Ehrlichia ruminantium)* in tropical Africa and is also a vector for *Rickettsia africae* and *Babesia divergens,* both of which are potential human pathogens [1,13]. The relevance of *A. variegatum* in relation to canine vector-borne disease is unclear: that particular dog was also parasitised by *R. sanguineus* and was positive for canine ehrlichiosis.

With the widespread presence of *R. sanguineus* ticks throughout the dog population, it was not surprising to find approximately one third of the dogs tested had antibodies to *Ehrlichia* spp. *Ehrlichia canis* has not previously been documented in Mauritius, although there is clinical suspicion amongst veterinarians and anecdotal evidence of its presence in the island (Ignace, unpublished observations) [2]. Canine monocytic ehrlichiosis results in a multi-systemic disease in dogs with clinical signs that range from mild to life-threatening (e.g. terminal myelosuppressive CME) [16]. The relatively high prevalence of ehrlichial infection in the study population should alert veterinarians to the likely importance of the disease with regards to canine health in Mauritius. The diagnostic test used in this study (Snap 4Dx Plus, IDEXX laboratories, Westbrook, ME, USA) does not differentiate between *E. canis* and *Ehrlichia ewingii*, and unfortunately we were unable to investigate this further by molecular testing. *Ehrlichia ewingii* is believed to be transmitted by *Amblyomma americanum* in the southern USA and the lone star tick has never been documented in Mauritius [17]. However, there are reports of *E. ewingii* in Cameroon and Brazil, which are other regions not known to be enzootic for *A. americanum* [18,19], potentially suggesting a different vector for this pathogen and we are therefore unable to confidently exclude its presence in Mauritius.

Approximately 15% of the dogs were seropositive for antibodies to *Anaplasma*, which could either represent *Anaplasma phagocytophilum* or *Anaplasma platys* infections (Snap 4Dx Plus, IDEXX laboratories, Westbrook, ME, USA). *Anaplasma phagocytophilum* is the cause of granulocytic anaplasmosis, infecting neutrophils of the canine host, and manifests clinically as non-specific signs of disease such as lameness, lethargy, pyrexia [20]. This member of the Anaplasmataceae is transmitted by *Ixodes* spp. ticks, which have not been previously identified on dogs in Mauritius, and their northern hemisphere distribution makes this form of anaplasmosis unlikely to occur in the island. A more plausible explanation for these results is infection by *A. platys* [21]. *Anaplasma platys* causes canine infectious cyclic thrombocytopenia (CICT) and may further complicate the pathogenesis of *E. canis*; both pathogens are generally found in similar geographical locations, both are transmitted by the same tick species, and it is common to find dogs concurrently infected with both pathogens (5% of the dogs in our study were infected with both *A. platys* and *E. canis*) [21].

*Borrelia burgdorferi*, the causative agent of Lyme disease, is transmitted by *Ixodes* spp. ticks. Clinical signs in dogs include lameness from inflammation of joints, lethargy and loss of appetite [22]. To the best of our knowledge autochthonous cases of Lyme disease have never been documented in Mauritius and with the presumed absence of the documented *Ixodes* spp. vectors, it is not believed to be present in the island. None of the dogs tested in this study returned a positive antibody test to the C6 antigen, a specific and highly conserved antigen expressed by members of the *Borrelia burgdorferi* genogroup *sensu lato*.

Hepatozoonosis in dogs is caused by two species; *H. americanum* and *H. canis*. To date, *H. americanum* has only been found in the United States and is transmitted by *Amblyomma maculatum* in that country. *H. canis* causes a much milder disease (anaemia, lethargy and often subclinical) and is transmitted by *R. sanguineus* throughout the world [23,24]. Despite causing mild disease, *H. canis* was found to be associated with lower PCV values in our dog population (p=0.03). This may be explained by the fact that only two dogs had single infections with *H. canis*, and that lower PCV values may actually be due to co-infections with other pathogens.

Hepatozoonosis is more difficult to diagnose as serological testing is not readily available for the causative pathogen, and either blood smears or PCR are required instead for diagnosis. The white cells were able to be visualised clearly by microscopy and *H. canis* gamonts were seen within the neutrophils of nine dogs. This is the first record of canine *Hepatozoon* infection in Mauritius.

*Dirofilaria immitis* is the cause of heartworm disease and is transmitted by mosquitoes. It has a worldwide distribution. Clinical signs include coughing, lethargy and exercise intolerance but some dogs have subclinical disease. The test used in this study detects *D. immitis* antigen from mature female worms only, therefore, a positive result indicates infection with at least one mature female heartworm (>6 months old) [21]. Interestingly 7 out of the 14 antigen positive dogs (50%; 95% CI 23, 77) were amicrofilaraemic on examination of blood smears, indicating the presence of occult infections in these dogs [25]. High percentages of occult infections are not uncommon in endemic areas and have previously been reported in central Portugal [26,27]; such infections have obvious diagnostic implications as clinicians must be careful when ruling out heartworm disease on the basis of microscopic work alone. Regardless, visualising microfilariae in blood smears is an insensitive way of quantifying the disease burden and should not be used as a singular method of diagnosis.

Curiously, one of the dogs was negative for the *D. immitis* antigen, but had visible microfilariae on microscopic examination. This may be explained by a false negative serological result (sensitivity of 99.0%- SNAP 4Dx Plus, IDEXX laboratories, Westbrook, ME, USA), or the dog was infected with a different filarial organism. Other filarial organisms that could present with microfilariae include, and are not limited to, *Dirofilaria repens*, *Acanthocheilonema reconditum*, *Acanthocheilonema dracunculoides*, *Brugia malayi*, *Brugia pahangi* [28]. Results from Chi-square testing also revealed a statistically significant relationship between lower tick burdens and infection with *D. immitis* infection. This could be coincidental or may reflect the fact that the dogs came from various parts of the island where mosquitoes with heartworm may be more prevalent in areas where ticks are less numerous.

A previous study carried out by Gaunt *et al.* showed that dogs co-infected with both *E. canis* and *A. platys* had more severe anaemia and thrombocytopenia, and a more persistent *A. platys* infection with a stronger immune response [29]. In our study, the number of pathogens the dogs were infected with did not have any statistically significant effect on PCV values, indicating that co-infected dogs did not show greater levels of anaemia. However, it must be acknowledged that PCV is not necessarily a reliable indicator of the degree of pathology for the vector-borne diseases we tested for, and that thrombocyte count may be more useful, but this would have required more sophisticated equipment. Furthermore, the unavailability of molecular analysis of the blood samples, such as by PCR, is acknowledged as a limitation of this diagnostic study, and this deficit should be addressed in any future studies of CVBD on Mauritius.

The relationship between tick burdens and infection rates was also assessed. Studies have shown that high tick burdens do not correlate with mortality or infection from tick-borne disease because not all ticks harbour pathogens [30,31]. This, however, was not the case with our study: our results showed that higher tick burdens were associated with *E. canis* and *H. canis* infections, as well as higher co-infection rates. This appears to be logical, as the more ticks a dog is infected with, the higher the chances of it contracting vector-borne pathogens.

## Conclusions

This is the first study to investigate the presence of canine vector-borne diseases in Mauritius. Our preliminary data confirm the presence of canine ehrlichiosis, anaplasmosis, hepatozoonosis and heartworm disease within the island, and show that dogs are at a high risk of harbouring these pathogens. Additional research based on molecular methods is required for evaluating the presence of *Babesia* spp., especially *B. c. vogeli* as its vector *R. sanguineus* is known to be present on the island. A more thorough link between haematological parameters and clinical presentation would also strengthen our knowledge about the vector-borne diseases on the island. This study also confirms the presence of *R. sanguineus* and *A. variegatum* in the dog population, with *R. sanguineus* being the most common tick parasitising dogs. High tick burdens were associated with greater infections from tick-borne diseases.
